# Personalized Treatment Suggestions: The Validity and Applicability of the Risk-Prevention-Index Social in Low Back Pain Exercise Treatments

**DOI:** 10.3390/jcm9041197

**Published:** 2020-04-22

**Authors:** Pia-Maria Wippert, Anne-Katrin Puschmann, David Drießlein, Winfried Banzer, Heidrun Beck, Marcus Schiltenwolf, Christian Schneider, Frank Mayer

**Affiliations:** 1 Sociology of Health and Physical Activity, University of Potsdam, 14469 Potsdam, Germany; apuschma@uni-potsdam.de; 2 Laboratory of Movement Biomechanics, Department of Health Sciences and Technology, ETH Zurich, 8092 Zurich, Switzerland; 3 Statistical Consulting Unit StaBLab, Ludwig-Maximilians-Universität München, 80539 Munich, Germany; david.driesslein@gmail.com; 4 Department of Sports Medicine, Goethe University Frankfurt, 60323 Frankfurt am Main, Germany; banzer@med.uni-frankfurt.de; 5 University Hospital Carl Gustav Carus at Technical University Dresden, 01307 Dresden, Germany; Heidrun.Beck@uniklinikum-dresden.de; 6 Pain Management, Centre of Orthopaedics and Trauma Surgery, Heidelberg University Hospital, 69118 Heidelberg, Germany; marcus.schiltenwolf@med.uni-heidelberg.de; 7 Orthopädiezentrum Theresie, 80339 München, Germany; dr.schneider@oz-theresie.de; 8 University Outpatient Clinic, Centre of Sports Medicine, University of Potsdam, 14469 Potsdam, Germany; fmayer@uni-potsdam.de

**Keywords:** back pain diagnosis, pain screening, exercise treatment, yellow flags

## Abstract

**Background:** The back pain screening tool Risk-Prevention-**I**ndex **S**ocial (RPI-S) identifies the individual psychosocial risk for low back pain chronification and supports the allocation of patients at risk in additional multidisciplinary treatments. The study objectives were to evaluate (1) the prognostic validity of the RPI-S for a 6-month time frame and (2) the clinical benefit of the RPI-S. **Methods:** In a multicenter single-blind 3-armed randomized controlled trial, *n* = 660 persons (age 18–65 years) were randomly assigned to a twelve-week uni- or multidisciplinary exercise intervention or control group. Psychosocial risk was assessed by the RPI-S domain social environment (RPI-S_SE_) and the outcome pain by the Chronic Pain Grade Questionnaire (baseline M1, 12-weeks M4, 24-weeks M5). Prognostic validity was quantified by the root mean squared error (RMSE) within the control group. The clinical benefit of RPI-S_SE_ was calculated by repeated measures ANOVA in intervention groups. **Results:** A subsample of *n* = 274 participants (mean = 38.0 years, SD 13.1) was analyzed, of which 30% were classified at risk in their psychosocial profile. The half-year prognostic validity was good (RMSE for disability of 9.04 at M4 and of 9.73 at M5; RMSE for pain intensity of 12.45 at M4 and of 14.49 at M5). People at risk showed significantly stronger reduction in pain disability and intensity at M4/M5, if participating in a multidisciplinary exercise treatment. Subjects at no risk showed a smaller reduction in pain disability in both interventions and no group differences for pain intensity. Regarding disability due to pain, around 41% of the sample would gain an unfitted treatment without the back pain screening. **Conclusion:** The RPI-S_SE_ prognostic validity demonstrated good applicability and a clinical benefit confirmed by a clear advantage of an individualized treatment possibility.

## 1. Introduction

Low back pain (LBP) is one of the most disabling health complaints with the world´s highest disability rate [1], causing immense costs for the health care systems [2]. Evidence-based treatment guidelines for LBP [3,4] point out that individual psychosocial factors, such as distress, pain-related cognitions or social environment, play a major role in the chronification of unspecific LBP [5,6,7]. Some Cochrane Reviews conclude that physical or medical therapy treatments are more successful if they are combined with psychosocial elements as multimodal therapy in comparison to unimodal therapy [8,9,10,11].

To date, however, it has not been conclusively clarified why, and which type of multidisciplinary therapy can achieve a better effect. After all, such effects can depend on the individual risk profile of a person on the one hand and on the reinforcing mechanisms of action of different therapy modules on the other. Regarding exercise therapy, there are indications that psychosocial factors may influence physiological exercise adaptation outcomes [12,13,14,15]. For example, it has been shown that the individual distress level influences the exercise dose-response effect on pain and depression outcomes, whereby a high exercise dose leads to worse outcomes because of additional high stress levels [16,17]. These rarely analysed mediation or moderation effects of the relationship to exercise adaption [18] could offer an explanation for the often-reported short-lasting effects of exercise treatments on LBP reduction [19]. Besides this, selection tools to detect individual risk profiles and to find the right combination and dose of exercise and behavioral treatment are lacking to the greatest extent. The most commonly used screening instruments evaluate psychosocial risk factors for LBP chronicity for primary care and without suggestions for an individualized treatment allocation [20,21,22,23]. Solely the Keele StarT Back (SBT [4,24]) provides an individual risk profile identification and allocation to uni- or multimodal treatment for physiotherapy and usual care in primary prevention, But this is not similarly applicable for exercise treatments. Recently, the RPI-S was developed, a screening tool that focuses on psychosocial risk and protective factors with respect to exercise treatments (e.g., individual distress vs. social support) [25]. The instrument was developed for a 1-year prognosis in a secondary prevention setting, and it is still unknown whether the RPI-S is valid for a shorter prediction in another population, and whether its applicability and benefit for clinical practice is acceptable.

The objective of this study is an explorative evaluation of the RPI-S, 1) whether the RPI-S is valid for a half-year prognosis within a secondary prevention sample and 2) whether the use of RPI-S results in an appropriate individual treatment allocation to uni- or multidisciplinary exercise treatment and clinical benefit. This should be analysed exemplarily for one risk domain of the RPI-S, whereby it is assumed that individuals at risk are more responsive to a synergistic LBP multidisciplinary therapy than people at no risk due to possible interaction effects.

## 2. Material and Methods

### 2.1. Design and Procedure

Study objectives were pursued in a subsample of a multicentre single-blind 3-armed randomised controlled trial (RCT, trial No. DRKS00004977, study design see [26,27] within the MiSpEx network (https://mispex.de/machbarkeit-multimodales-training/). The 24-week study comprised five measurements: baseline (M1), after 3 weeks (M2), after 6 weeks (M3), after 12 weeks (M4) and after 24 weeks (M5), whereby only results related to M4 and M5 were reported here. Each measurement started with a questionnaire administered by a study nurse and was followed by a medical examination. At baseline, subjects were randomly assigned (n_block_ = 18, basis 1:1; www.randomization.com) to an exercise intervention (**S**ensori**M**otor **T**raining; SMT), a multidisciplinary exercise intervention (**S**ensori**M**otor **T**raining and **B**ehavioral **T**raining; SMT+BT), and a control group (regular routines) [28]. Subjects in the intervention groups participated in a supervised centre-based training of 3 weeks, followed by 9 weeks of home-based training, with an average duration of 30 minutes 3 times/week. Study personnel were blinded and participants were told not to communicate their group allocation to other participants or staff.

All clinical investigations have been conducted according to the principles expressed in the Declaration of Helsinki. Final ethical approval has been provided 01/25/2012 by the major institutional ethics review board of the University of Potsdam, Germany (number 36/2011). The study was registered as a clinical trial 05/16/2013 in the German Clinical Trial Register with the identification number: DRKS00004977. Study conduction was between 06/2013-12/2014. Adverse events were reported in the manuscript.

### 2.2. Intervention

The exercise intervention included four different sensorimotor exercises, of which two directly trained the core stabilizing and core surrounding muscles, while the other two indirectly trained upper and/or lower extremities (detailed description see [27,29,30]). At the centre-based phase, the subjects were guided by sports- and physiotherapists, while the home training was audio-guided by a DVD. Therapists provided support by phone, e-mail or face-to-face contact for all participants at any time. The additional Behavioral-Therapy module (BT module) included an education film, cognitive distraction tasks (while practicing sensorimotor training) and body scan tasks provided by a DVD (for detailed description see [27]). The educational film (to be watched with the life partner) focussed on the social environment of the participant’s inclusive partnership task and three units: I) an *education phase* (explanation of social and personal risk factors and the bio-psycho-social model of chronic pain), II) an *exercise phase* (coping strategies and tasks), and III) a *transfer phase* (reflection on and improvement of daily life routines).

### 2.3. Participants

Participants (*n* = 744) between 18–65 years were recruited at different clinical study sites across Germany. Low back pain appearance (lowest ribs to gluteal folds) was defined as a minimum pain intensity score of 20 on a 100-point Visual Analogue Scale. Sample size calculation was based on a feasibility study that yielded an effect size for the outcome variable pain of *f* = 0.25 (*α* ≤ 0.05; 1-*β* = 0.999, drop out 30%, power analysis by G*Power [31]).

The inclusion criteria were: at least one episode (≥4 days) of nonspecific low back pain in the previous 12 months, to be able to understand the extent and content of the study and to answer a German questionnaire without help. The exclusion criteria were pregnancy, acute pain during the past seven days, being unable to stand upright, not able to give outcome sick leave information, or showing signs of acute risk factors referred to as “red flags” (inflammatory, traumatic or systematic processes). All participants signed a written informed consent after receiving written and oral information about the study (Flow chart of Enrolment see [27] and S1).

The recruited participants follow the same inclusion and exclusion criteria as participants within the developmental study of the RPI-S [25]. For this reason, sample characteristics are similar.

### 2.4. Instruments

The randomized controlled multicenter trial contained a broad measurement set up (such as questionnaires for the assessment of stress, depression, anxiety, fear-avoidance, pain vigilance, social support/attachment and lifestyle) which is not described in detail here, because the manuscript is focusing on the quality of the Risk Prevention Index (RPI-S, e.g., [29,32]).

#### 2.4.1. Outcome Variables

Pain was assessed by the Chronic Pain Grade questionnaire (CPG [33]), a seven-item questionnaire assessing pain intensity and disability, during the intervention period on a 10-point Likert scale. Scoring reveals two subscales “characteristic pain intensity” (CPI) and “subjective pain disability” (DISS) ranging from 0 to 100. Further, subjects can be graded due to the severity of their pain (0—no pain complaints to IV—high disability). Internal consistency (Cronbach´s alpha) in the sample was 0.85 for CPI and 0.91 for DISS.

#### 2.4.2. Prognostic Variables

The individual psychosocial risk profile for getting chronic low back pain and the expected treatment response were assessed at baseline (M1) by one domain of the RPI-S [25], which is a screening tool that should facilitate physician’s decision making in back pain management. The instrument screens for the individual risk constellation in four psychosocial risk domains: subjective distress, pain experiences, social environment, and medical care environment related to each outcome (pain intensity CPI and pain disability DISS). The relevant items for the RPI-S were selected by Lasso algorithm [25] to obtain a total score for each domain and outcome, ranging from 0 to 100, item scores were weighted by regression coefficients and summed up. The analyses for clinical benefit were performed only for the social **e**nvironment domain of the RPI-S (RPI-S domain social environment (RPI-S_SE_)_,_ total 20 items, of which 15 items scores for CPI and 17 for DISS), because not all RPI-S domain-variables were included in this randomized controlled trial. A score above the domain-specific critical cut-off would indicate the need of an additional treatment in the respective domain, which means that a multidisciplinary exercise treatment strategy would be recommended. For subjects with low domain scores, a unimodal exercise treatment would be recommended. The cut-offs for people at higher risk were already determined in the development sample by ROC curves, in which the RPI-S reached acceptable discriminant validity with AUC = 0.79 for DISS and AUC = 0.82 for CPI [25].

The development and validation of the back pain screening tool RPI-S was carried out along the PROGnosis RESearch Strategy [34] to investigate clinical outcomes in three consecutive multi-center longitudinal studies within the MiSpEx network [35] and has passed through all 4 PROGRES steps [36,37,38] with around 3500 participants to date. The presented study objectives treat a first (retrospective) external evaluation of the validity and clinical relevance as it is suggested for the 4th PROGRES step [38]; here, only one RPI-S domain was analyzed, because the development was not finalized at this stage. A further external validation with all RPI-S domains and a comparison to the STarTBack [24] was conducted in a one-year two-armed randomized controlled multicenter trial (Trial No. DRKS 00010129, [30]) with 1600 participants, but not published yet. Finally, a further consecutively three-armed randomized controlled multicenter trial (Trial No. DRKS 00020373) with 1200 chronic low back pain patients has just started, in which the RPI-S then serves a priori for a therapy allocation.

#### 2.4.3. Statistics and Data Analysis

Study objectives were analyzed in a subsample of the presented RCT, whereby the subsample selection was based on complete cases (in RPI-S) and on subjects with a minimum CPG-Pain Class of 1. Both conditions were necessary for a strict validity check. The variable income was estimated from a comparable sample.

### 2.5. Validity and Prognosis Error

The prognosis error between predicted and observed values of CPI and DISS at 12-weeks and 24 weeks follow-up was calculated with the root mean squared error (RMSE [39]), using only the data of control subjects.

### 2.6. Responsiveness and Applicability

Subjects with high RPI-S_SE_ scores are expected to profit more from the multidisciplinary (SMT+BT) than from the unimodal exercise treatment (SMT), i.e., larger reductions of CPI and DISS. For subjects with low scores, a unimodal treatment should be ample. Due to the explorative character of the study (no a priori allocation to the treatment groups based on the risk scores), this analysis could only be performed retrospectively.

For the evaluation of the clinical benefit, the sample was split alongside the cut-off scores of the RPI-S_SE_ in both intervention groups. Subjects with an RPI-S_SE_ score of ≥31 (CPI) and RPI-S_SE_ ≥ 17 (DISS) were classified as the medium/high risk group (defined as at risk), whereas subjects with RPI-S_SE_ < 31 (CPI) and RPI-S_SE_ < 17 (DISS) were classified as the low risk group (defined as at no risk). For each of the two risk groups, repeated measures analyses of variances were calculated for CPI and DISS at baseline (M1), 12 weeks follow-up (M4, after homebased intervention), and 24 weeks follow-up (M5, 3 months after the end of intervention) (factor: *time*) comparing the unimodal (SMT) and multidisciplinary (SMT+BT) intervention groups (factor: intervention *group*) and their development over time (interaction: time*intervention group). Simple contrasts were used to compare pain values at each of the follow-up measurements with baseline pain. The significance was set at *p* < 0.05. Uncorrected *p*- values were reported due to the explorative character of the analyses. Analysis was only performed in the highly standardized intervention groups.

## 3. Results 

### 3.1. Descriptives 

In total, *n* = 660 volunteers were randomly allocated in the randomized controlled trial (see S2). A subsample of *n* = 274 persons fulfilled the study criteria for the analysis presented here, which were a pain class higher zero (CPG > 0) and complete cases for the RPI-S_SE_ for M1, M4 and M5. These participants showed a mean age of 37.94 years, with a standard deviation of *SD* = 13.08 and a moderate characteristic pain intensity (CPI) at baseline (M1): *M* = 34.08 (*SD* = 17.44), at 12 weeks follow-up (M4): *M* = 26.21 (*SD* = 17.83) and at 24 weeks follow-up (M5): *M* = 24.84 (*SD* = 17.61); disability (DISS) improved from baseline (M1): *M* = 19.98 (*SD* = 21.98), to 12 weeks follow-up (M4): *M* = 10.41 (*SD* = 15.58) and to 24 weeks follow-up (M5): *M* = 8.49 (*SD* = 13.21). In total, 49.8% of respondents lived in partnership, 42.5% were single and 7.7% widow/divorced/separated. A total of 17.5% were still in education, 22.6% had a low, 20.1% a medium and 36.1% a high level of education. With regard to network contacts, the respondents had an average of *M* = 5.22 (*SD* = 3.47) family members in constant contact and an average of 10.39 (*SD* = 8.04) friends in their circle of friends. The frequency of contact with the close reference person is described by 84% as intensive. Work absence due to pain was on average *M* = 2.35 (*SD* = 10.14) days within the last 3 months at baseline. Most of these variables were not related to the outcome development. Only marital status shows a low correlation with M4 and M5 for both outcomes (Pearson between *r* = 0.19 and 0.27, *p* < 0.001). 

Baseline RPI-S_SE_ scores were *M* = 25.34 (*SD* = 11.65) related to CPI and *M* = 13.40 (*SD* = 9.66) related to DISS. The distribution of the sample within people at risk and no risk by the RPI-S_SE_ cut-off is presented in Table 1. Differences in DISS and CPI between baseline and M4 and M5 for the RPI-S_SE_ groups are displayed in Table 2.

### 3.2. Validity and Prognosis Error

The results for the root mean squared error (RMSE) within the control group are presented in Table 3. The second column shows high prognostic validity for a half year with more accuracy than the prediction of future pain outcomes on baseline values.

### 3.3. Responsiveness and Applicability

#### Characteristic Pain Intensity

Repeated measures ANOVA revealed a significant main effect of *time* in both risk groups (at no risk: *Fc*(2, 196) = 9.577, *p* < 0.001, *η_part_²* = 0.089; at risk: *F*(2, 94) = 33.815, *p* < 0.001, *η_part_²* = 0.418), meaning that, regardless of group allocation, CPI changed significantly from M1 to follow-up. The effect was medium in people at no risk and strong for people at risk. Planned contrasts showed that this reduction in CPI is evident in both intervention groups from baseline to M4 as well as from baseline to M5.

The main effect of the *intervention group* did not reach significance in either of both risk groups (at no risk: *F* (1, 98) = 1.253, *p* = 0.266, *η_part_²* = 0.013; at risk: *F* (1, 47) = 0.125, *p* = 0.725, *η_part_²* = 0.003), which means that an overall group difference between SMT and SMT+BT was not detected.

**Figure 1:**
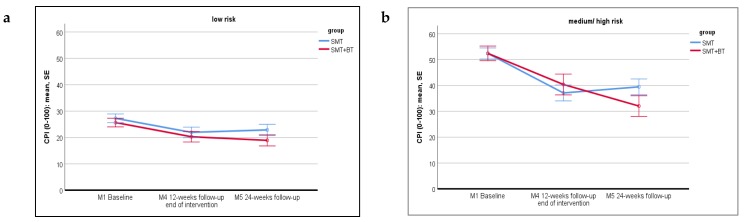
Displaying the time * group interaction for CPI in the ‘at-no-risk’ (low risk **a**) and ‘at-risk’ (medium/ high risk, **b**) group, comparing SMT vs. SMT+BT at the three measurements.

There is only a significant time*intervention group interaction for people at risk (*F* (2, 94), *p* = 0.044, *η_part_²* = 0.064; people at no risk: *F* (2, 196) = 0.435, *p* = 0.648) (see Figure 1a,b). People at risk benefit from a larger reduction in CPI from baseline to M5 in the SMT+BT (*M* = -21.75, *SD* = 19.34) than participants in the SMT group (*M*= -12.93, *SD* = 14.48), although contrast analysis does not reach significance (*F* (1, 47) = 2.704, *p* = 0.107).

### 3.4. Disability

Repeated measures ANOVA reveals a significant main effect of *time* in both groups (at no risk: *F* (2, 134) = 6.568, *p* = 0.002, *η_part_²* = 0.089; at risk: *F* (2, 58) = 27.453, *p* < 0.001, *η_part_²* = 0.486), meaning that, regardless of group allocation, DISS changes significantly in both groups from M1 to both follow-ups. The effect is strong for persons at risk and medium for persons at no risk.

Furthermore, there is no significant main effect of *intervention group* in both risk groups (no risk: *F* (1, 67) = 0.235, *p* = 0.630, *η_part_²* = 0.003; at risk: *F* (1, 29) = 0.755, *p* = 0.392, *η_part_²* = 0.025), meaning that an overall difference between SMT and SMT+BT is not detected.

**Figure 2:**
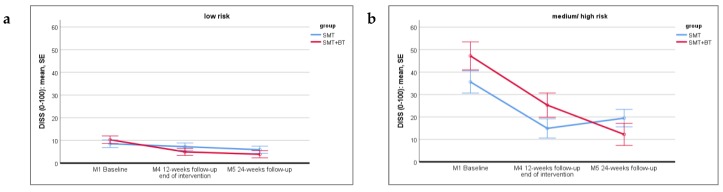
Displaying the time * group interaction for DISS in the at-no-risk (low risk **a**) and at-risk (medium/ high risk **b**) group, comparing SMT vs. SMT+BT at the three measurements.

Finally, there is a significant time*intervention group interaction within the at-risk group: *F* (2, 58), *p* = 0.022, *η_part_²* = 0.123 (see Figure 2a,b). Planned contrasts show that persons at risk benefit from a larger reduction in DISS from baseline to M5 in the SMT+BT (*M* = -36.46, *SD* = 23.99) than in the SMT group (*M*= -23.19, *SD* = 29.91).

## 4. Discussion

The objective of the study was an evaluation of the applicability and validity of the recently developed back pain screening tool RPI-S [25], which aims to facilitate individual treatment allocation decisions within an exercise treatment context. For this purpose, one RPI-S domain (namely RPI-S_SE_) was applied in a subsample of a 3-group RCT with a uni- and multidisciplinary exercise treatment.

Concerning the question of whether the Risk Prevention Index (RPI-S_SE_) is valid for a half-year prognosis in a secondary preventive context, it was shown that the prognosis errors within this study are better than those of the development sample [25] and furthermore exacter than those of a prediction on baseline values, confirming the applicability of the instrument.

Related to the question of whether the use of RPI-S_SE_ results in an appropriate allocation to unimodal or multidisciplinary exercise treatment, it was shown that individuals at risk in their social environment (high scores in the RPI-S_SE_), benefited more from the multidisciplinary treatment regarding the reduction of pain-related disability in comparison to individuals at no risk. It is important to note here that the observed improvements were of high sustainability, as this is an essential criteria in the evaluation of a therapy success [40]. This specific pattern could also be observed for self-reported characteristic pain intensity, although the effect was small and less specific. People at higher risk in RPI-S_SE_ consistently showed an improvement in pain intensity, although this could not be clearly attributed to a specific form of intervention.

The last reported result may reflect the general variability of self-reported characteristic pain intensity over time due to, for example, specific and non-specific treatment effects [41,42]. In contrast, pain-related disability more often represents chronic states that strongly impact social tasks, and is itself influenced by the individual’s environment [43]. The offered education with the spouse addresses maladaptive pain-cognitions (e.g., fear, avoidance beliefs), responsible medication intake and a stimulation of adaptive interactions with the social environment. This may help to improve disability states.

Regarding participants at risk in the presented sample, it is appropriate to conclude that 41% regarding disability and 38% regarding self-reported characteristic pain intensity were treated with a unimodal exercise intervention, hence with an intervention that probably does not offer the best benefit for these individuals. On the other hand, 67% (for DISS) and 71% (for CPI) were probably ‘overtreated’ in the multidisciplinary group because, according to their RPI-S-risk score, the unimodal exercise treatment would have been sufficient. In total, 70% were classified as being at no risk, and initially would have to be allocated to unimodal treatment, and 30% were classified as being at risk and would therefore possibly benefit more from a multidisciplinary treatment option. The results refer to the risk due to the social environment and its habits for the persons under study. Information about the other risk domains (e.g., stress, pain experience, medial environment) cannot be derived from the data and will be examined in the follow-up study.

The Risk Stratification Index (domain RPI-S_SE_) determine the prevalence of risk groups similar to the StarT Back [24], which classifies 28% of high-risk individuals with a need for multidisciplinary treatment and 72% of no-risk individuals with back pain of any duration with allocation to unimodal treatment with physiotherapy only. The RPI-S, in general, may have a different sensitivity to previous psychosocial screening tools, which was recently criticized [44,45], but this has to be examined further.

## 5. Limitations

There are some limitations to respect: (1) in this explorative study, only one risk domain (RPI-S_SE_) was evaluated, which does not allow a transfer to other RPI-S domains; (2) the evaluation was only retrospectively analysed, therefore missing an a priori design; (3) the sample size is limited due to complete cases and CPG > 0 selection, which leads to small groups in the final analysis. (4) The term multidisciplinary intervention was used for our combined SMT+BT tool; this is different to a multidisciplinary form in clinics, which requires an agreement between different experts.

## 6. Conclusions

The validity and clinical benefit of the RPI-S domain social environment was proven in a half-year randomized controlled trial with a uni- and multidisciplinary approach. Individuals assigned “at risk” by the RPI-S_SE_ benefited more from a multidisciplinary exercise treatment than subjects “at no risk”. The RPI-S is the first LBP screening tool that can be used in the context of exercise treatments. It further gives information about four psychosocial risk domains (mainly yellow flags), which provides practitioners with more specific knowledge about their patient´s individual risk profiles and treatment needs. Applying the RPI-S may prevent the over- or under-treatment of patients due to unspecific alignment to treatment options. Nevertheless, further developmental and validation steps are strongly required. Therefore, a further external validation [30] and a priori stratification by the RPI-S is planned in two consecutively randomized controlled multicentre trials.

## Figures and Tables

**Table 1:** Numbers of subjects within the intervention groups stratified into ‘at risk’ and ‘at no risk’ groups by the RPI-S domain social environment (RPI-S_SE_).

RPI-S_SE_ Group		RPI-S_SE_	Intervention Group			Total	
			CG	SMT	SMT+BT		
**DISS**	at no risk	< 17	49	44	42	135	69.2%
	at risk	≥ 17	8	31	21	60	30.8%
		Total	57	75	63	195	100.0%
**CPI**	at no risk	< 31	60	67	64	191	70.0%
	at risk	≥ 31	15	41	26	82	30.0%
		Total	75	108	90	273	100.0%

CG—control group, SMT—unimodal sensorimotor training, SMT+BT—multimodal sensorimotor training combined with behavioral therapy modules, CPI—characteristic pain intensity, DISS—disability score, RPI-S_SE_—RPI-S domain social environment.

**Table 2:** Differences in DISS and CPI between baseline (M1), 12 weeks follow-up (M4) and 24 weeks follow-up (M5). Negative values indicate a reduction at M4 and M5, respectively.

	**Characteristic Pain Intensity—CPI**							
	**At no risk**				**at risk**			
	**ΔCPI**_**M4M1**_		**ΔCPI**_**M5M1**_		**ΔCPI**_**M4M1**_		**ΔCPI**_**M5M1**_	
	**mean**	**sd**	**mean**	**sd**	**mean**	**sd**	**mean**	**sd**
CG	–6.48	12.33	–6.24	12.52	–12.14	15.45	–10.48	19.74
SMT	–3.77	16.27	–4.17	19.10	–14.44	14.54	–12.93	14.48
SMT+BT	–4.94	11.70	–6.76	13.10	–16.94	21.44	–21.75	19.34
	**Disability-Score—DISS**							
	**At no risk**				**at risk**			
	**ΔDISS**_**M4M1**_		**ΔDISS**_**M5M1**_		**ΔDISS**_**M4M1**_		**ΔDISS**_**M5M1**_	
	**mean**	**sd**	**mean**	**sd**	**mean**	**sd**	**mean**	**sd**
CG	–3.03	7.04	–1.96	8.09	–25.00	16.33	–26.19	18.20
SMT	–2.00	9.57	–2.19	12.09	–18.78	28.79	–23.19	29.91
SMT+BT	–5.70	9.10	–5.56	11.43	–30.19	26.95	–36.46	23.99

CG—control group, SMT—unimodal sensorimotor training, SMT+BT—multimodal sensorimotor training combined with behavioural therapy modules, CPI—characteristic pain intensity, DISS—disability score.

**Table 3:** Predictive validity of RPI-SSE for CPI and DISS using RMSE, compared to prediction on the baseline level of CPI and DISS.

	M4—Baseline RMSE	M4—Prediction RMSE	*n*	M5—Baseline RMSE	M5—Prediction RMSE	*n*
CPI	15.09	12.45	68	16.03	14.49	62
DISS	13.45	9.04	52	14.23	9.73	48

RMSE—root mean squared error, CPI—characteristic pain intensity, DISS—disability score, n—observation numbers (may differ due to differences in completion of DISS and CPI at M4 and M5).

## Supplementary Materials

The following are available online at https://www.mdpi.com/2077-0383/9/4/1197/s1; S1: Consort Flow Diagram.

## Abbreviations

AUC	Area under the Curve
CPG	Chronic Pain Grade Questionnaire
CPI	Characteristic Pain intensity, scale of Chronic Pain Grade questionnaire
DISS	subjective pain disability, scale of Chronic Pain Grade questionnaire
DRKS	German Clinical Trials Register
*p* -values	significance level: *p* <.01, *p* <.05 or *p* <.10
LBP	Low Back Pain
M1, M2, M3, M4, M5	visits 1-5
*N*	number of participants
MiSpEx	the German Research Network of Medicine in Spine Exercise
RMSE	root mean squared error (= prognosis error between predicted value and real observed values).
RPI-S	Risk Prevention Index – Social
RPI-S_SE_	Risk Prevention Index – Social: domain social environment
SBT	Keele StarT Back
SMT	sensorimotor training (=motor control exercise)
SMT+BT	sensorimotor training with behavioral therapy elements

## References

[1] Hoy D.G., Bain C., Williams G., March L., Brooks P., Blyth F., Woolf A., Vos T., Buchbinder R. (2012). A systematic review of the global prevalence of low back pain. Arthritis Rheum.

[2] Wenig C.M., Schmidt C.O., Kohlmann T., Schweikert B. (2009). Costs of back pain in Germany. Eur. J. Pain.

[3] Foster N.E., Mullis R., Hill J.C., Lewis M., Whitehurst D.G.T., Doyle C., Konstantinou K., Main C., Somerville S., Sowden G. (2014). Effect of Stratified Care for Low Back Pain in Familiy Practice (IMPaCT Back): A Prospective Population-Based Sequential Comparison. Ann. Fam. Med..

[4] Hill J.C., Whitehurst D.G.T., Lewis M., Bryan S., Dunn K.M., Foster N.E., Konstantinou K., Main C.J., Mason E., Somerville S. (2011). Comparison of stratified primary care managment for low back pain with current best practice (STarTBack): A randomised controlled trial. Lancet.

[5] Airaksinen O., Brox J.I., Cedraschi C., Hildebrandt J., Klaber-Moffett J., Kovacs F.M., Mannion A.F., Reis S., Staal J.B., Ursin H. (2006). Chapter 4. European guidelines for the management of chronic nonspecific low back pain. Eur. Spine J..

[6] Nicholas M.K., Linton S.J., Watson P.J., Main C.J. (2011). Early identification and management of psychological risk factors (“yellow flags”) in patients with low back pain: A reappraisal. Phys. Ther..

[7] Main C.J., Kendall N.A., Hasenbring M., Hasenbring M., Rusus A.C., Turk D.C. (2012). Screening of psychosocial risk factors (yellow flags) for chronic back pain and disability. From Acute to Chronic Back Pain: Risk Factors, Mechanisms and Clinical Implications.

[8] Engers A.J., Jellema P., Wensing M., Van Der Windt D., Grol R., Van Tulder M. (2008). Individual patient education for low back pain. Cochrane Database Syst. Rev..

[9] Hayden J.A., Van Tulder M., Malmivaara A., Koes B.W. (2005). Exercise therapy for treatment of non-specific low back pain. Cochrane Database Syst. Rev..

[10] Henschke N., Ostelo R., Van Tulder M., Vlaeyen J.W., Morley S., Assendelft W., Main C.J. (2010). Behavioural treatment for chronic low-back pain. Cochrane Database Syst. Rev..

[11] Kamper S., Apeldoorn A.T., Chiarotto A., Smeets R.J., Ostelo R., Guzman J., Van Tulder M. (2014). Multidisciplinary biopsychosocial rehabilitation for chronic low back pain. Cochrane Database Syst. Rev..

[12] Picard M., McManus M.J., Gray J.D., Nasca C., Moffat C., Kopinski P.K., Seifert E.L., McEwen B.S., Wallace D.C. (2015). Mitochondrial functions moudlate neuroendocrine, metabolic, inflammatory and transcriptional responses to acute psychological stress. Proc. Natl. Acad. Sci. USA.

[13] Picard M., Russel T.H., Burelle Y. (2012). Mitochondrial functional specialization in glycolytic and oxidative muscle fibers: Tailoring the organelle for optimal function. Am. J. Physiol. Cell Physiol..

[14] Wippert P.M., Wiebking C. (2016). Adaptation to physical activity and mental stress in the context of pain: Psychobiological aspects. Schmerz.

[15] Wippert P.M., Wiebking C. (2018). Stress and Alterations in the Pain Matrix: A Biopsychosocial Perspective on Back Pain, its Prevention and Treatment. Int. J. Environ. Res. Public Health.

[16] Cook D.B., Stegner A.J., Ellingson L.D. (2010). Exercise alters pain sensitivity in Gulf War veterans with chronic musculoskeletal pain. J. Pain.

[17] McEwen B.S., Kalia M. (2010). The role of corticosteroids and stress in chronic pain conditions. Metabolism.

[18] Puschmann A.-K., Drießlein D., Beck H., Arampatzis A., Catalá M.M., Schiltenwolf M., Mayer F., Wippert P.-M. (2020). Stress and self-efficacy as long-term predictors for chronic low back pain: A prospective longitudinal study. J. Pain Res..

[19] Saragiotto B.T., Maher C., Yamato T.P., Costa L.O.P., Costa L.D.C.M., Ostelo R., Macedo L.G. (2016). Motor control exercise for chronic non-specific low-back pain. Cochrane Database Syst. Rev..

[20] Neubauer E., Junge A., Pirron P., Seemann H., Schiltenwolf M. (2006). HKF-R 10—screening for predicting chronicity in acute low back pain (LBP): A prospective clinical trial. Eur. J. Pain.

[21] Stiefel F., De Jonge P., Huyse F.J., Guex P., Slaets J.P., Lyons J.S., Spagnoli J., Vannotti M. (1999). *“*INTERMED”: A method to assess health service needs. II. Results on its validity and clinical use. Gen. Hosp. Psychiatry.

[22] Traeger A., Henschke N., Hübscher M., Williams C.M., Kamper S., Maher C., Moseley G.L., McAuley J.H. (2016). Estimating the Risk of Chronic Pain: Development and Validation of a Prognostic Model (PICKUP) for Patients with Acute Low back pain. PLoS Med..

[23] Lentz T., Beneciuk J., Bialosky J.E., Zeppieri G., Dai Y., Wu S.S., George S.Z. (2016). Development of a yellow flag assessment tool for orthopaedic physical therapists: Results from the optimal screening for prediction of referral and outcome (OSPRO) cohort. J. Orthop. Sports Phys..

[24] Hill J.C., Dunn K.M., Lewis M., Mullis R., Main C.J., Foster N.E., Hay E.M. (2008). A primary care back pain screening tool: Identifying patient subgroups for initial treatment. Arthritis Rheum.

[25] Wippert P.-M., Puschmann A.-K., Drießlein D., Arampatzis A., Banzer W., Beck H., Schiltenwolf M., Schmidt H., Schneider C., Mayer F. (2017). Development of a Risk Stratification and Prevention Index for stratified care in chronic low back pain. Focus: Yellow flags (MiSpEx Network). Pain Rep..

[26] Mayer F., Arampatzis A., Banzer W., Beck H., Brüggemann G.-P., Hasenbring M., Kellmann M., Kleinert J., Schiltenwolf M., Schmidt H. (2018). Medicine in Spine Exercise [MiSpEx]—A National Research Network to evaluate Back Pain in High-performance Sports as well as the General Population. Z. Sportmed..

[27] Wippert P.-M., Drießlein D., Beck H., Schneider C., Puschmann A.-K., Banzer W., Schiltenwolf M. (2020). The Feasibility and Effectiveness of a New Practical Multidisciplinary Treatment for Low-Back Pain: A Randomized Controlled Trial. J. Clin. Med..

[28] Hönning A., Stengel D., Güthoff C. (2018). Statistical strategies to address main research questions of the MiSpEx network and meta-analytical approaches. Ger. J. Sports Med..

[29] Wippert P.M., de Witt Huberts J., Klipker K., Gantz S., Schiltenwolf M., Mayer F. (2015). Development and content of the behavioral therapy module of the MiSpEx intervention: Randomized, controlled trial on chronic nonspecific low back pain. Schmerz.

[30] Niederer D., Vogt L., Wippert P.-M., Puschmann A.-K., Pfeifer A.-C., Schiltenwolf M., Banzer W., Mayer F. (2016). Medicine in spine exercise (MiSpEx) for nonspecific low back pain patients: Study protocol for a multicentre, single-blind randomized controlled trial. Trials.

[31] Faul F., Erdfelder E., Lang A.-G., Buchner A. (2007). G* Power 3: A flexible statistical power analysis program for the social, behavioral, and biomedical sciences. Behav. Res. Methods.

[32] Von Korff M., Ormel J., Keefe F.J., Dworkin S.F. (1992). Grading the severity of chronic pain. Pain.

[33] Hemingway H., Riley R.D., Altman D.G. (2009). Ten steps towards improving prognosis research. BMJ.

[34] Wippert P.-M., Arampatzis A., Banzer W., Beck H., Hasenbring M.I., Schiltenwolf M., Schneider C., Stengel D., Platen P., Mayer F. (2019). Psychosoziale Risikofaktoren in der Entstehung von chronisch unspezifischen Rückenschmerzen: Auszug aus der methodischen Rationale der Multicenterstudien in MiSpEx. Z. Sportpsychol..

[35] Riley R.D., Hayden J.A., Steyerberg E.W., Moons K.G.M., Abrams K., Kyzas P.A., Malats N., Briggs A., Schroter S., Altman U.G. (2013). Prognosis Research Strategy (PROGRESS) 2: Prognostic factor research. PLoS Med..

[36] Steyerberg E.W., Moons K.G.M., Van Der Windt D.A., Hayden J.A., Perel P., Schroter S., Riley R.D., Hemingway H., Altman D.G. (2013). Prognosis Research Strategy (PROGRESS) 3: Prognostic model research. PLoS Med..

[37] Hingorani A., A Van Der Windt D., Riley R.D., Abrams K., Moons K.G.M., Steyerberg E.W., Schroter S., Sauerbrei W., Altman U.G., Hemingway H. (2013). Prognosis research strategy (PROGRESS) 4: Stratified medicine research. Br. Med. J..

[38] Rüschendorf L. (2014). Mathematische Statistik.

[39] Schiltenwolf M., Buchner M., Heindl B., Von Reumont J., Müller A., Eich W. (2006). Comparison of a biopsychosocial therapy (BT) with a conventional biomedical therapy (MT) of subacute low back pain in the first episode of sick leave: A randomized controlled trial. Eur. Spine J..

[40] Von Korff M., Turk D.C., Melzack R. (2011). Assessment of chronic pain in epidemiological and health services research. Handbook of Pain Assessment.

[41] Whitney C.W., von Korff M. (1992). Regression to the mean in treated versus untreated chronic pain. Pain.

[42] Wippert P.M., Fliesser M., Krause M. (2017). Risk and protective factors in the clinical rehabilitation of chronic back pain. J. Pain Res..

[43] Karran E., McAuley J.H., Traeger A., Hillier S., Grabherr L., Russek L.N., Moseley G.L. (2017). Can screening instruments accurately determine poor outcome risk in adults with recent onset low back pain? A systematic review and meta-analysis. BMC Med..

[44] Karran E., Traeger A., McAuley J.H., Hillier S., Yau Y.-H., Moseley G.L. (2017). The value of prognostic screening for patients with low back pain in secondary care. J. Pain.

[45] Kongsted A., Andersen C.H., Hansen M.M., Hestbaek L. (2016). Prediction of outcome in patients with low back pain—a prospective cohort study comparing clinicians predictions with those of the STarT Back Tool. Man. Ther..

